# Excess cardiovascular mortality associated with cold spells in the Czech Republic

**DOI:** 10.1186/1471-2458-9-19

**Published:** 2009-01-15

**Authors:** Jan Kysely, Lucie Pokorna, Jan Kyncl, Bohumir Kriz

**Affiliations:** 1 Institute of Atmospheric Physics, Academy of Sciences of the Czech Republic, Bocni II 1401, 141 31 Prague, Czech Republic; 2 Centre for Epidemiology and Microbiology, National Institute of Public Health, Prague, Czech Republic; 3 Department of Epidemiology, Third Faculty of Medicine, Charles University in Prague, Czech Republic

## Abstract

**Background:**

The association between cardiovascular mortality and winter cold spells was evaluated in the population of the Czech Republic over 21-yr period 1986–2006. No comprehensive study on cold-related mortality in central Europe has been carried out despite the fact that cold air invasions are more frequent and severe in this region than in western and southern Europe.

**Methods:**

Cold spells were defined as periods of days on which air temperature does not exceed -3.5°C. Days on which mortality was affected by epidemics of influenza/acute respiratory infections were identified and omitted from the analysis. Excess cardiovascular mortality was determined after the long-term changes and the seasonal cycle in mortality had been removed. Excess mortality during and after cold spells was examined in individual age groups and genders.

**Results:**

Cold spells were associated with positive mean excess cardiovascular mortality in all age groups (25–59, 60–69, 70–79 and 80+ years) and in both men and women. The relative mortality effects were most pronounced and most direct in middle-aged men (25–59 years), which contrasts with majority of studies on cold-related mortality in other regions. The estimated excess mortality during the severe cold spells in January 1987 (+274 cardiovascular deaths) is comparable to that attributed to the most severe heat wave in this region in 1994.

**Conclusion:**

The results show that cold stress has a considerable impact on mortality in central Europe, representing a public health threat of an importance similar to heat waves. The elevated mortality risks in men aged 25–59 years may be related to occupational exposure of large numbers of men working outdoors in winter. Early warnings and preventive measures based on weather forecast and targeted on the susceptible parts of the population may help mitigate the effects of cold spells and save lives.

## Background

Morbidity and mortality of a population are influenced by a large number of factors, one of them being meteorological conditions. In mid-latitudes, the most direct effects of weather on human health are observed during and after summer heat waves [1-8]. Cold-related mortality, although much less understood than heat-related mortality, is another example of the effects of weather on the human health. Increases in mortality with decreasing temperatures in winter have been reported in many regions, mainly in mid-latitudes [1,9-15]. The lags between cold weather and its impacts on mortality are usually longer (about a week compared with 0–1 days for heat-related mortality), and the relationship is less direct, geographically more variable, and it may be partly confounded by other factors that strongly influence mortality in winter (e.g. influenza epidemics and outbreaks of other acute respiratory infections).

The effects of cold weather are usually most apparent in mortality due to cardiovascular and respiratory diseases [16,17] and in the elderly [e.g. [1,10,15]]. After heat waves, a harvesting effect often occurs for several days, but cold spells may rise levels of mortality for several weeks and no subsequent declines of mortality rates below expected levels have been observed [16].

Cold-related mortality has been examined relatively comprehensively in western and southern Europe [10,13,15,17-19], but there have been few studies in central-European populations. This is somewhat surprising, taking into account the fact that central Europe experiences colder winters and more severe cold spells, owing to the stronger influences of continental high-pressure systems (supporting low air temperatures in winter) and invasions of cold air from north and northeast (which are often blocked or moderated by the Alps). In addition, a different political and socio-economic development, with economic transformation starting in the early 1990s, and generally less prosperous conditions and lower socio-economic and health status of the population contribute to the specificity of the central-European region.

The present paper examines the effects of winter cold spells on cardiovascular mortality in the population of the Czech Republic, with particular attention to their impact on different population groups and to the lags of mortality effects. We focus on the population of the whole country (between 10.20 and 10.36 million over the entire period 1986–2006), since the sample is much larger than those for the city of Prague and other large cities, and more robust conclusions may be derived (only a small part of the population – 21% – lives in cities with more than 100 000 inhabitants [20]). The area of the Czech Republic is relatively small (78.9 thousand km^2^), which makes it possible to use average temperature series calculated from a large set of meteorological stations (see Section Methods) to describe daily variations in weather.

## Methods

### Daily mortality data, removal of effects of influenza/ARI epidemics

Daily data on mortality due to cardiovascular diseases (CVD; ICD-9 codes 390–459, 1986–1993; ICD-10 codes I00–I99, 1994–2006) in the Czech Republic over 1986–2006, stratified by gender and age groups (0, 1–4, 5–14, 15–24, 25–59, 60–69, 70–79, and 80+ years), were provided by the Institute of Health Information and Statistics. (Note that the term 'mortality' is used for numbers of deaths and not mortality rates throughout the paper.) The younger age groups (up to 24 years) were not involved in the analysis owing to very small sample sizes in cardiovascular mortality data. Mortality due to CVD accounted for 49.9% and 59.5% of total mortality in men and women, respectively, over the examined 21-yr period, representing by far the most frequent cause of death. Only the main cause of death was considered. The percentage of death certificates that are based on autopsy (30.2% in 2006) is relatively large in the Czech Republic and similar to some western European countries [21], which supports reliability of the database.

**Figure 1:**
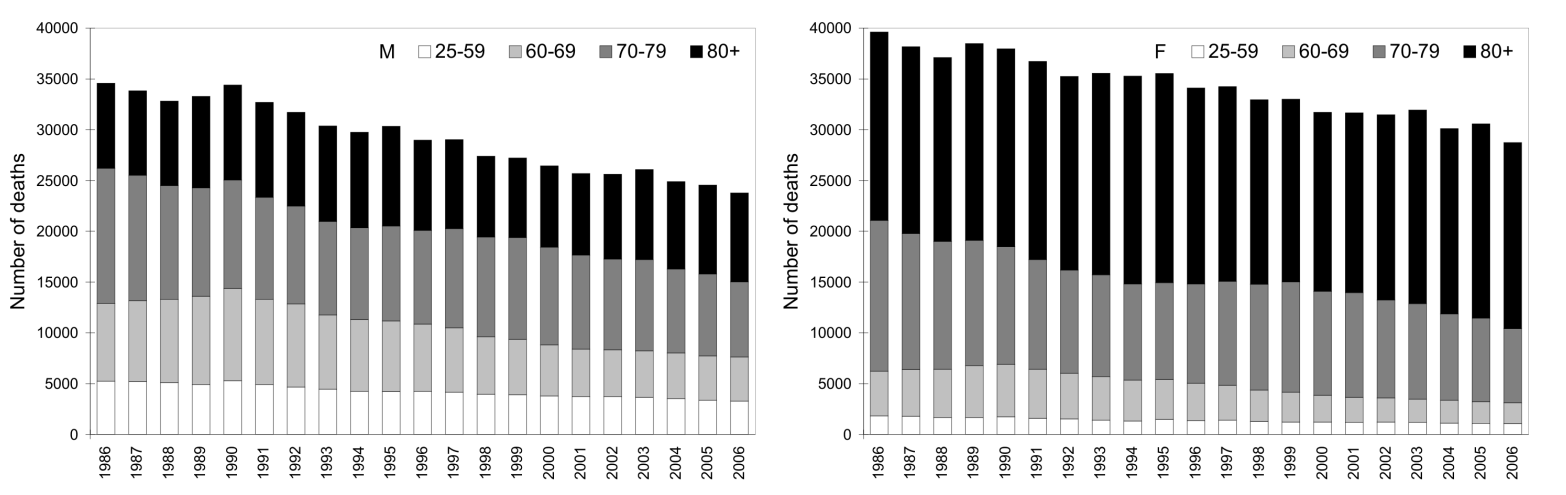
**Annual numbers of deaths from cardiovascular diseases by age and sex during the period 1986–2006**. M (F) stands for men (women).

Since long-term changes, due to demographic, medical-technological as well as life-style changes, and a seasonal cycle are manifested in the mortality data, the daily death counts had to be standardized. We used a procedure similar to that applied in other studies, e.g. in [5,22-24]. This approach consists in estimating excess daily mortality by calculating deviations of the observed number of deaths and the expected (baseline) number of deaths for each day of the examined period. The expected number of deaths takes into account the long-term changes in mortality (related to enhanced life expectancy after socio-economic changes that followed the collapse of communism in 1989; Figure 1) as well as short-term variations due to the seasonal cycle (larger mortality in late than early winter).

**Figure 2:**
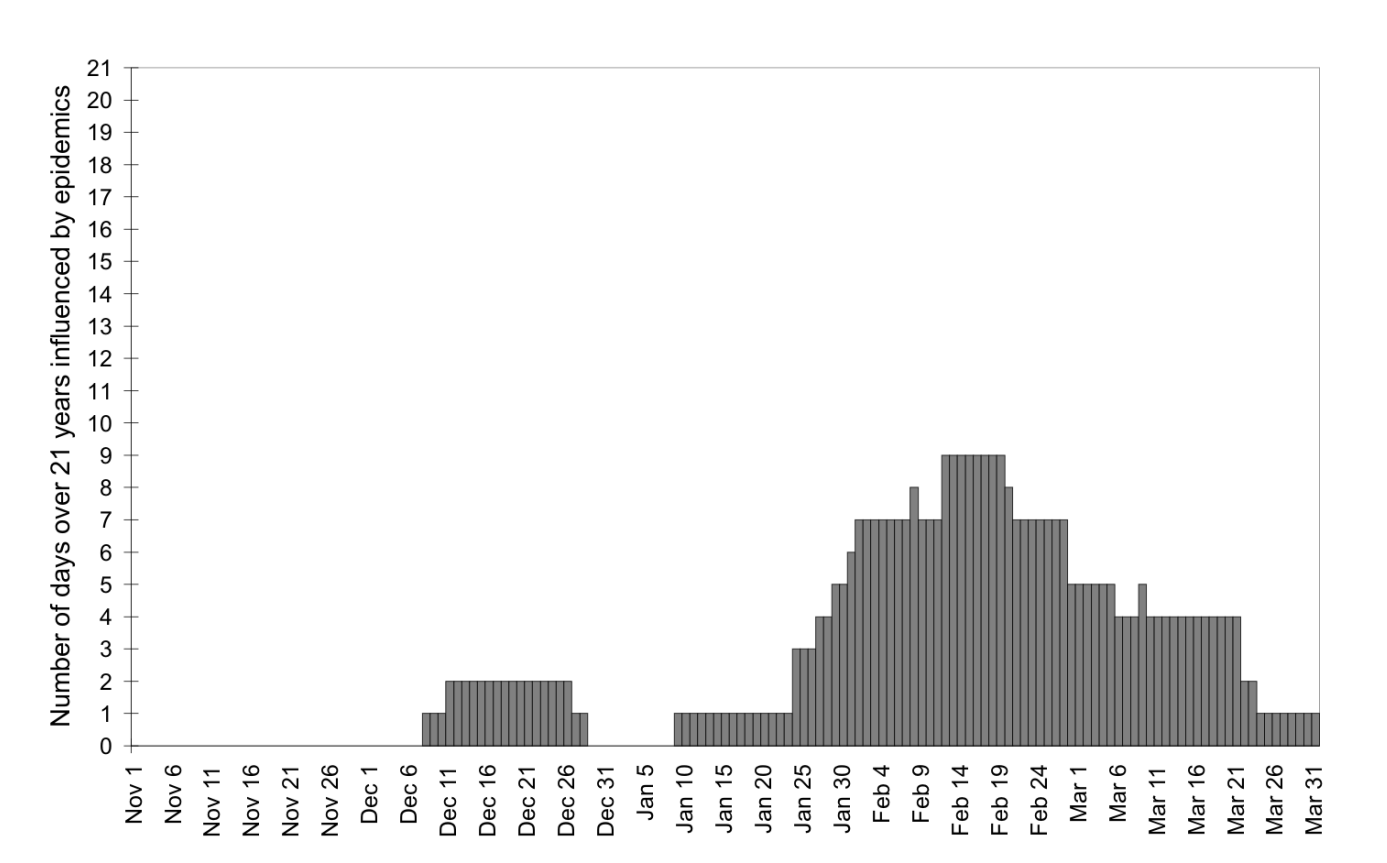
**Numbers of days over the November-March period, 1986–2006, on which mortality was affected by influenza epidemics**.

Before calculating the expected mortality, epidemics of influenza and acute respiratory infections (ARI) had been identified and excluded from the data from which the mean annual cycle was determined; in this procedure, a dataset on morbidity from influenza/ARI was taken from the national surveillance system, and the national epidemic threshold of 2000 reported ill per 100000 population was applied [25]. (ARI for reporting purposes is defined as every general practitioner's clinical diagnosis of acute upper respiratory tract infection [as defined by the International Classification of Diseases, ICD-10 codes J00, J02, J04, J05, J06] and influenza [ICD-10 codes J10.1, J10.8, J11.1, J11.8].) 13 epidemics covering altogether 399 days were identified over 1986–2006; all occurred in the December-March period, with a peak in February (Figure 2). The relationships between influenza/ARI and mortality were found to be strongest with the 7-day lag of mortality after morbidity (for both genders), which is consistent with a previous study [26]. This 7-day lag was applied always when the effects of the epidemics were considered. The exact procedure was as follows: 1) All days in epidemics were identified. 2) All days corresponding to the epidemics, with the 7-day lag, were removed from the analysis. Seasonal variations in the numbers of days excluded from the analysis because of the epidemics are shown in Figure 2.

Employing this approach, the expected number of deaths *M*_0_(*y*, *d*) for year *y *(*y *= 1986, ... 2006) and day *d *(*d *= 1, ... 365) was set according to

M_0_(y, d) = M_0_(d).Y(y)

where *M*_0_*(d) *denotes the mean daily number of deaths on day *d *in a year (computed from the mean annual cycle smoothed by 15-day running means), and *Y(y) *is a correction factor for the observed year-to-year changes in mortality, defined as a ratio of the number of deaths in year *y *to the mean annual number of deaths during the analyzed period. Correction factors for the year-to-year changes *Y(y) *were calculated over the April-November period when data are not confounded by the epidemics of influenza/ARI.

Standardization of the mortality data according to the above given formula was applied for each age group (including 'all ages' group) and gender separately.

The 95% confidence intervals for excess mortality were calculated using the lower and upper limit factors for a Poisson-distributed variable according to [27]; for the number of cases larger than 100, the normal approximation was used.

### Meteorological data

**Figure 3:**
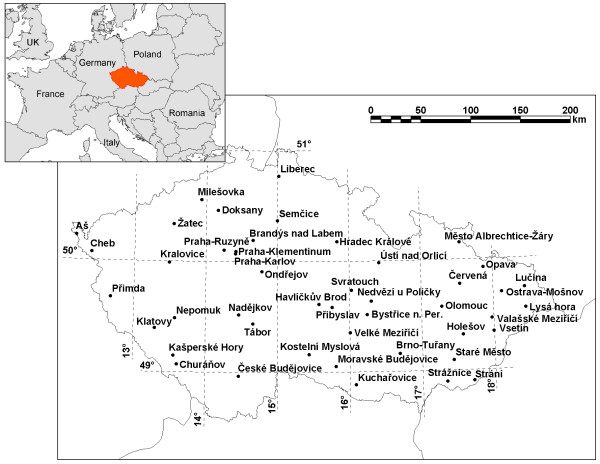
**Area under study and locations of meteorological stations**.

Daily series of maximum (TMAX) and minimum (TMIN) air temperatures were computed as averages over 46 high-quality stations operated by the Czech Hydrometeorological Institute and covering the area of the Czech Republic (Figure 3). The stations were selected according to three basic criteria: (i) the series at all stations are complete, (ii) no important station moves that could result in inhomogeneities in the records occurred over 1986–2006, and (iii) the spatial coverage is relatively even and the average air temperature series are representative for the population under study.

### Cold spells

There is no operational definition of cold spells in central Europe, and various approaches appear in the literature. For example, Domonkos et al. [28] defined extreme cold events in south-central Europe using the threshold of daily average temperature -5.0°C. We tested several possible definitions of cold spells, and decided to define them as periods of at least 3 consecutive days with daily air temperature maxima (TMAX) < -3.5°C in the average series for the Czech Republic. The threshold value is a useful compromise between possible thresholds that lead to too few (although very severe) cold spells on the one hand, and those that delimit a relatively large number (but often little pronounced) cold events on the other. 10 days with TMAX below the chosen threshold occur during an average winter season, which makes the examined sample relatively wide; in each year, at least one day with TMAX < -3.5°C was recorded, with maximum values in winters 1986/87 (28 days) and 1995/96 (23 days). The definition leads to 28 cold spells over 21 years, with their mean duration of 5.0 days (4 out of 28 cold spells were completely removed from the analysis due to epidemics). No decreasing trend in the frequency of cold spells was observed over the 21-yr period.

A preliminary analysis also showed that relationships between excess mortality and cold spells are better expressed with this relatively low threshold compared to possible higher thresholds that yield more frequent cold spells.

The definition based on TMAX was applied because of somewhat stronger links to excess mortality than those for daily air temperature minima (TMIN); this is in accord with the results for Madrid [15]. Also, TMAX is a measure of daytime ambient temperature exposure which captures the thermal stress better and influences more on most parts of the population than night-time air temperature. However, as a tentative rule, days with low TMAX are characterized by low TMIN, too. Cold spells according to the introduced definition occur in November-March, so only data over this period enter the analysis.

## Results

Lagged relationships between excess mortality and cold spells were examined separately for total population (all ages) and individual age groups 25–59, 60–69, 70–79 and 80+ years. The lag is always considered on a daily scale, not from the beginning or the end of a cold spell; if D1 (Dn) denotes the first (last) day of a cold spell, the period with lag *x *is simply the interval D1+*x*, Dn+*x*. The lags of mortality impacts after cold spells up to 20 days were evaluated, and for each age group, the lag at which the excess mortality was most pronounced (averaged over all days in cold spells over 1986–2006) was determined (Table 1). Cold spells with at least one day on which mortality was affected by an epidemic of influenza/ARI (with the 7-day lag after morbidity, see Section Methods) were excluded from the analysis.

**Table 1:** Lags of excess mortality after cold spells in individual population groups and characteristics of excess CVD mortality associated with cold spells.

Population group	Lag with the largest mean relative excess CVD mortality [days]	Relative mean excess CVD mortality with the lag after cold spells and 95% CI [%]	Mean excess CVD mortality with the lag after cold spells and 95% CI [number of deaths]	Mean expected CVD mortality [number of deaths]	Percentage of cold spells with positive excess CVD mortality at the given lag [%]
All ages, M	2	6.3 (4.2; 8.3)	5.4 (3.6; 7.1)	85.4	75.0
All ages, F	9	6.3 (4.4; 8.2)	6.5 (4.6; 8.5)	103.4	87.5

25–59 yrs, M	1	13.8 (8.4; 19.1)	1.7 (1.0; 2.3)	12.1	87.5
25–59 yrs, F	2	6.9 (-2.5; 17.4)	0.3 (-0.1; 0.7)	4.0	70.8

60–69 yrs, M	10	3.8 (-0.6; 8.3)	0.7 (-0.1; 1.5)	18.0	56.5
60–69 yrs, F	2	7.5 (1.9; 13.6)	0.8 (0.2; 1.4)	10.3	62.5

70–79 yrs, M	5	6.4 (3.1; 9.9)	1.9 (0.9; 2.9)	29.1	66.7
70–79 yrs, F	11	7.5 (4.1; 10.8)	2.3 (1.3; 3.4)	31.4	82.6

80+ yrs, M	4	8.5 (5.0; 12.2)	2.2 (1.3; 3.2)	26.1	80.0
80+ yrs, F	9	7.3 (4.9; 9.7)	4.2 (2.8; 5.6)	57.7	83.3

M (F) stands for men (women). Mean values are calculated as averages over days in cold spells with the given lag of mortality.

### Basic features of excess CVD mortality associated with cold spells

The mean relative excess CVD mortality associated with cold spells was positive for all age groups and in both men and women (Table 1). Rather surprisingly, the most pronounced mortality effects appeared in men aged 25–59 years, with the 1-day lag after weather (+13.8%; 95% CI 8.4–19.1%). Positive excess mortality in this population group occurred in 21 out of 24 cold spells (the other 4 cold spells were excluded due to epidemics). In no other age group, and in neither men nor women, the mean relative increase in CVD mortality exceeded 8.5%, a value much smaller than the 13.8% increase in men 25–59 years. The percentage of cold spells with positive excess CVD mortality (at the lags shown in Table 1) was between 57 and 88%; the percentage was very large also in women when all age groups were considered together (88%). The lags of the most pronounced mortality effects were longer in women than men, except for the age group 60–69 years in which the mean relative excess mortality in women was largest for the lag of 2 days.

Differences between average excess CVD mortality associated with cold spell days (with the lags as shown in Table 1) and on the other days unaffected by influenza/ARI epidemics in winter season (November-March) were statistically significant at p = 0.01 in all population groups except for women 25–59 and men 60–69 years (Table 2). The differences in the whole population made 5.5 CVD deaths daily in men and 6.7 CVD deaths daily in women. Very similar results are obtained if differences in average *raw *mortality (instead of *excess *mortality) are evaluated as in [15]; however, the comparison of excess mortality is more suitable owing to the seasonal cycle and long-term trend present in baseline mortality. The estimated mean excess CVD mortality on the other days unaffected by epidemics and/or cold spells was close to zero in all population groups (Table 2), which supports plausibility of the standardization procedure and the baseline mortality estimates.

**Table 2:** Significance of the difference between mean excess CVD mortality associated with cold spells and on the other days in November-March excluding epidemics, for the same age groups and lags as in Table 1.

Population group	Mean excess CVD mortality associated with cold spell days [deaths/day]	Mean excess CVD mortality on other days in winter (Nov.-March) [deaths/day]	p value of the t-test for the difference in means
All ages, M	5.4	-0.2	< 0.001
All ages, F	6.5	-0.2	< 0.001

25–59 yrs, M	1.7	0.0	< 0.001
25–59 yrs, F	0.3	0.0	0.178

60–69 yrs, M	0.7	-0.1	0.083
60–69 yrs, F	0.8	-0.1	0.009

70–79 yrs, M	1.9	0.1	0.001
70–79 yrs, F	2.3	0.2	< 0.001

80+ yrs, M	2.2	-0.2	< 0.001
80+ yrs, F	4.2	-0.3	< 0.001

Two sample t-test was used for testing the difference in means.

### Mortality in men 25–59 years

**Figure 4:**
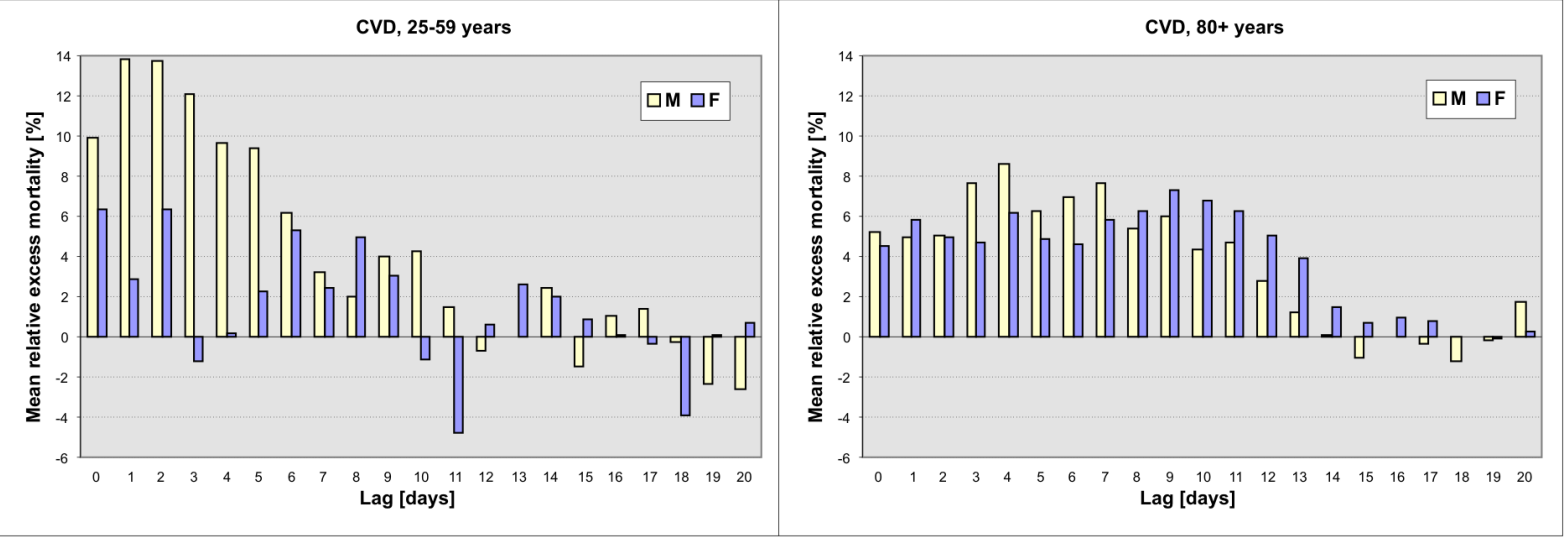
**Mean relative excess CVD mortality with the lag of 0 to 20 days after cold spells**. Left: men (M) and women (F) 25–59 years; right: M and F 80+ years.

Compared to any other population group, the mortality effects of cold stress were more direct and immediate in men 25–59 years; the relationship is strongest with the lag of 1 day, and the effects are confined to the period of up to several days after cold spells (Figure 4 left), unlike in the elderly in which elevated mortality (more than +5% above the baseline) persists for many days (up to 12 (13) days in men (women) for age group 80+ years; Figure 4 right). The link between cold spells and mortality is strongest at longer lags of 2 up to 11 days in all other population groups (Table 1).

### Mortality during and after the most severe cold spell in January 1987

Large increases in CVD mortality in the most susceptible group (men 25–59 years) as well as in the other population groups, mainly in men, were observed in January 1987 when the most severe cold spell occurred (Jan. 10–18). It was characterized by extremely low day-time air temperatures, with daily *maxima *below -10°C if averaged over the days in the cold spell, and -17.6°C on the peak day; it is worth noting that daily temperature maxima are usually above zero in the area under study even in the coldest months of year.

**Figure 5:**
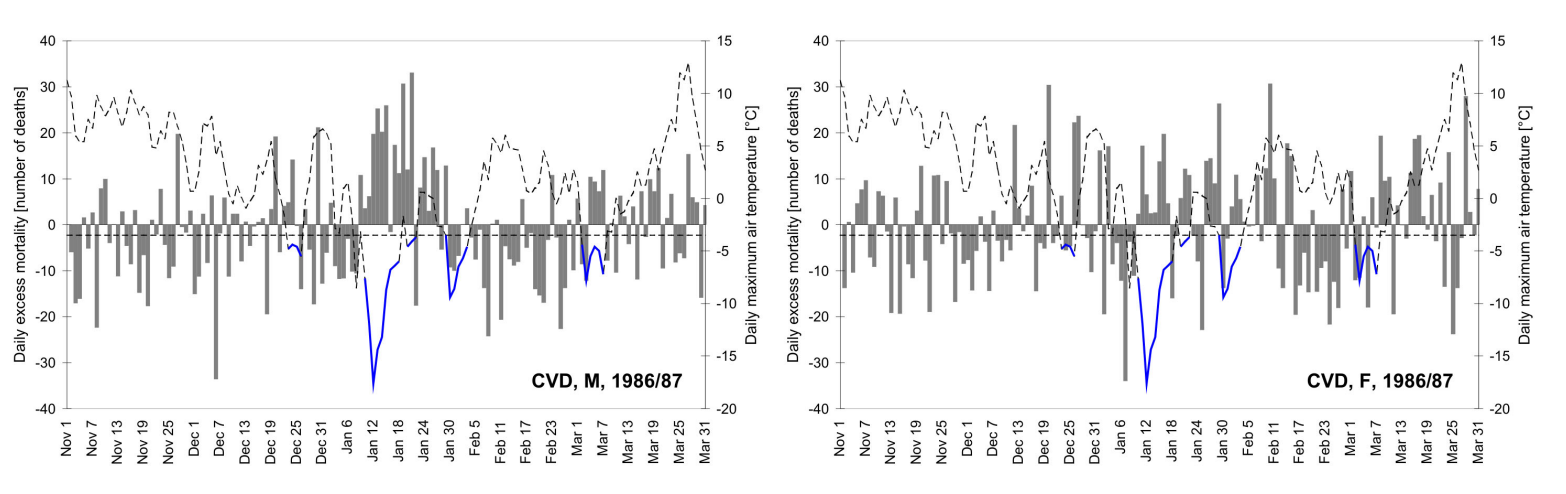
**Daily absolute excess CVD mortality (numbers of deaths, columns) and maximum daily air temperature (dashed curve) during the 1986/87 winter season**. Cold spells are marked with the blue colour. Left: men, right: women.

The course of daily temperature and excess CVD mortality during the 1986/87 winter season is plotted in Figure 5 separately for men (left) and women (right). Even this simple comparison reveals that the mortality impacts in men were better pronounced, more direct and occurring mostly during the cold spells, while the effects in women were somewhat less clear and more lagged. Mortality in men was above the baseline estimate for a consecutive 13-day period of Jan. 9–21 (except for Jan. 16), with the total number of +215 extra deaths due to CVD. Excess mortality in women over the same period was much smaller, +59 excess CVD deaths, and it does not become more pronounced if the lag of about 9 days (reported in Table 1) is taken into account. Note that the very first cold spell in the 1986/87 season (Dec. 23–26) was not evaluated in this study as it occurred during an influenza epidemic.

### Effects of influenza/ARI epidemics

**Figure 6:**
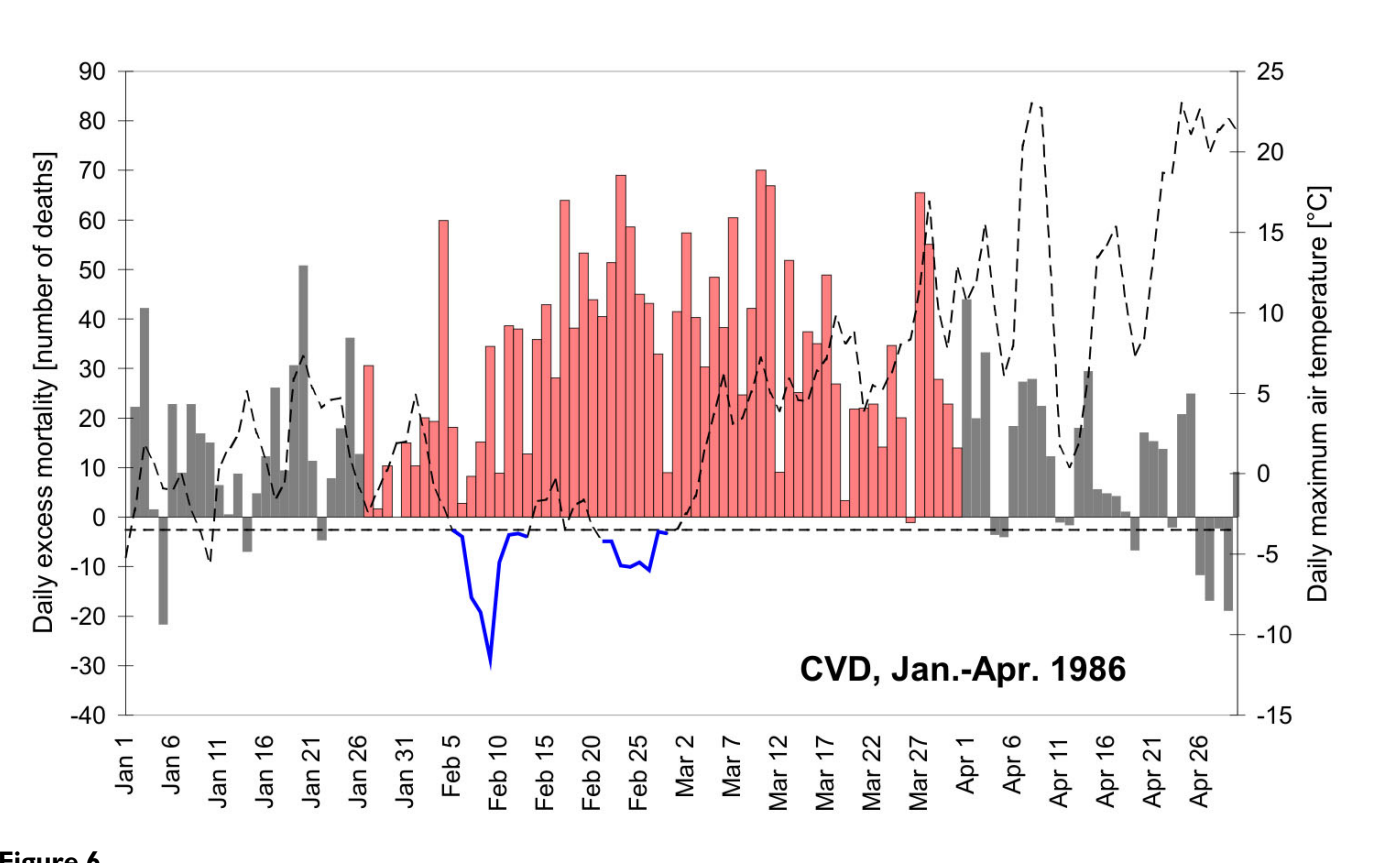
**Daily absolute excess CVD mortality (numbers of deaths, columns) and maximum daily air temperature (dashed curve) during January-April 1986**. Cold spells are marked with the blue colour, the influenza epidemic is plotted in red.

The effects of major influenza/ARI epidemics were controlled for in the analyzed mortality data, by considering an average lag of mortality effects after enhanced morbidity (7 days) and excluding the relevant data on mortality from the analysis. An example of excess mortality in cold spells that occurred during an influenza/ARI epidemic is shown in Figure 6 for the first months of 1986 when a severe epidemic hit the region (lasting for more than 2 months since the last decade of January). Excess CVD mortality was positive over the whole duration of the epidemic (if mortality on all days is summed up, with the lag after morbidity of 7 days, there were 2078 estimated excess CVD deaths between Jan. 27 and March 31; 95% CI 1834–2326). The epidemic was unusually severe, which was also demonstrated by the fact that mortality had already been elevated before the onset of the epidemic in January (when morbidity was still slightly below the threshold). The cold spell recorded between Feb. 5 and 13, with very low minimum TMAX (-11.5°C), could falsely be linked to large excess mortality if the occurrence of the epidemic had not been taken into account: with the lag of 7 days after weather, the excess CVD mortality was +357 deaths (95% CI 265–453). Such a large value would severely affect also average statistics concerning mortality effects associated with cold spells, and would rank the cold spell as the worst one according to mortality impacts (Table 3). It is obvious that the cold spell could have slightly risen mortality levels but the excess mortality was primarily due to the influenza/ARI epidemic (although indirect weather-related effects may influence morbidity, too). More robust statistical measures like median would be less affected and should be recommended in studies of cold-related mortality that do not explicitly take into account epidemics of infectious diseases and other non-meteorological factors affecting mortality in winter.

**Table 3:** Cold spells with the largest absolute excess CVD mortality in the examined population (all ages).

a. Men		
Cold spell	Excess CVD mortality relative to baseline [%]	Absolute excess CVD mortality (95% CI) [number of deaths]

1987 Jan 10 – 1987 Jan 18	17.7	161 (98.3; 227.5)

1991 Feb 4 – 1991 Feb 7	13.9	54 (13.9; 97.2)

1995 Dec 28 – 1995 Dec 31	26.6	91 (51.7; 134.8)

1999 Dec 23 – 1999 Dec 25	20.6	48 (16.3; 82.8)

2004 Jan 3 – 2004 Jan 6	24.7	72 (36.2; 112.1)

		
b. Women		

Cold spell	Excess CVD mortality relative to baseline [%]	Absolute excess CVD mortality (95% CI) [number of deaths]

1991 Jan 31 – 1991 Feb 2	30.0	101 (61.1; 144.0)

1995 Dec 28 – 1995 Dec 31	13.6	57 (15.3; 101.6)

1999 Dec 23 – 1999 Dec 25	29.7	84 (48.1; 124.5)

2002 Dec 24 – 2002 Dec 26	16.4	47 (12.7; 85.6)

2004 Jan 3 – 2004 Jan 6	27.8	102 (61.0; 146.9)

The lags of mortality effects after weather are the same as in Table 1.

It is also worth noting that since excess mortality during influenza/ARI epidemics occurs mainly in the elderly [29], the results of this study are influenced less by possible unaccounted 'minor epidemics' and outbreaks of acute respiratory infections or varied lags of their mortality effects for the middle-aged group (most affected by cold-related CVD mortality) than the elderly.

## Discussion

### Mortality risks associated with cold spells

The results show that cold spells are associated with positive mean excess CVD mortality in all examined age groups 25–59, 60–69, 70–79 and 80+ years, and both in men and women. When total population (all ages) is considered, the mean relative excess CVD mortality due to cold spells is 6.3% in men (with the lag of 2 days) and 6.3% in women (with the lag of 9 days), which corresponds on average to 5.5 (6.7) excess deaths per day in men (women). Since the estimated excess mortality is always specific to a given lag, it represents a rather conservative estimate and the overall excess mortality attributable to cold spells may be underestimated. The total impact of cold on mortality is underestimated also because the threshold temperature applied in the definition of what constitutes a cold spell is relatively low, and cold-related mortality may start at higher temperatures.

### The most susceptible population: men 25–59 years

The fact that the largest effects of cold spells on CVD mortality (in relative terms) appear in middle-aged men contrasts with findings of previous studies on cold-related mortality in other regions, which usually report most important effects in the elderly [e.g. [1,10,15,19]]. However, elevated mortality among younger men has also been documented [30], and Tillett et al. [31] reported significant impacts of cold weather on mortality in the age group 40–64 years in the UK. The present results suggest that risks of cold-related CVD mortality in the middle-aged population group, and particularly men, should be revisited as this part of population appears to be vulnerable to the effects of cold stress. The very short lag at which the mortality increase in men 25–59 years is most pronounced (1 day after temperature) means that the effects of cold in middle-aged men are most direct and excess deaths occur on the days with the cold weather and very shortly after.

We interpret the large mortality risks in men 25–59 years as being related to occupational exposure: much higher percentage of men than women work outdoors and some of them are directly exposed to ambient temperatures in winter. The proportion of men on the total number of employees in industry, construction and agriculture has been relatively stable in recent years, representing approximately 92% for construction, 87% for mining and quarrying, 63% for the other sectors of industry, and 69% for agriculture and forestry [32]. The older age groups (which are formed mainly by retired persons) can more easily adapt their activities to current weather condition. Although indoor temperatures may play an important role in exposing persons to cold stress in winter, too, the relatively direct relationship with a short lag after cold stress points to the effects of a shorter-term exposure to very low outdoor temperatures. We also note that housing conditions of the population (with respect to protecting against cold) have been of a relatively good standard over the analyzed period, with large majority of houses being equipped with central heating. The possible role of outdoor occupation in inducing cold-related mortality deserves further investigation.

The threshold of 60 years was used to separate the middle-age population group from the elderly as it corresponds closely to the usual age of retirement in the Czech Republic over most of the examined period (only in the most recent past, the age of retirement has started to increase gradually).

### Within-season acclimatization to cold

In three winter seasons, more than two cold spells occurred. There was no clear pattern of decreased or increased mortality effects associated with the later cold spells throughout the population groups; sometimes the first cold spell was linked with the largest mortality increase (more often for men than women) while in some other cases a reversed pattern was observed. However, if all ages are considered together, excess CVD mortality was always largest in the first winter cold spell for men, which may be indicative of some level of within-season acclimatization to cold.

### Mechanisms of excess cardiovascular mortality due to cold

Exposure to cold may lead to direct cardiovascular stress due to changes in blood pressure, vasoconstriction, and increase in blood viscosity and levels of red blood cell count, plasma cholesterol, and plasma fibrinogen [33]. Skin cooling has been shown to increase systematic vascular resistance, heart rate and blood pressure. Cold can induce myocardial ischemia, acute myocardial infarction and sudden death [34,35]. The physiological effects of cold exposure are dealt with in more detail e.g. by Bøkenes et al. [36] in the elderly and Mercer et al. [37] in younger persons. In addition, alcoholic beverages can increase skin cooling due to peripheral vasodilatation that could contribute as an additional factor of undercooling (which may consequently result in a cardiovascular failure). The most vulnerable groups from this point of view are homeless people.

### The most severe cold spells and associated mortality

The excess CVD mortality during the invasion of extremely cold air in 1987, +274 (95% CI: 165–385) deaths over 13-day period Jan. 9–21, is comparable (in terms of the absolute number of deaths as well as the relative magnitude of increase) with excess CVD mortality observed during the most severe heat wave in this region (+385 excess CVD deaths over 18-day period July 22–Aug. 8, 1994; [38]). This is a surprising finding and points to the fact that the importance of cold stress has been underestimated. While the effects of heat stress have been investigated in detail for the population of the Czech Republic [5,38,39], much less attention has been paid to cold-related mortality and no systematic study on this topic has been carried out. In contrast to heat-related mortality, the excess mortality associated with the cold spells is more direct and more pronounced in men than women, most probably due to the occupational reasons as mentioned above.

## Conclusion

The study shows that cold stress has an important impact on excess cardiovascular mortality in all population groups. The relative risks associated with cold spells are larger and more immediate in middle-aged men (25–59 years; on average +13.8% increase in mortality with the 1-day lag after weather) than in the other population groups, which is likely related to the effects of occupational exposure. The findings can be used in regularly issued biometeorological forecasts and warnings that may be targeted on the particularly vulnerable parts of the population. The action may contribute to the mitigation of possible impacts of cold spells, save human lives and generally improve well-being status of the population.

## Competing interests

The authors declare that they have no competing interests.

## Authors' contributions

JK designed and coordinated the study, drafted the manuscript and participated in the statistical analyses. LP carried out most statistical analyses. JK acted as the leading person in the interpretation of epidemiological data. BK participated in the design and coordination of the study. All authors contributed to the interpretation of results, commented the draft and approved the final manuscript.

## Pre-publication history

The pre-publication history for this paper can be accessed here:


